# Outbreak Caused by Multidrug-Resistant *Mycobacterium Tuberculosis* with Unusual Combination of Resistance Mutations, Northern Argentina, 2006–2022

**DOI:** 10.3201/eid3103.241272

**Published:** 2025-03

**Authors:** Roxana Paul, Federico Lorenzo, Beatriz López, María Gabriela Alegre, David Couvin, Nalin Rastogi, Laura Pérez-Lago, Darío García de Viedma, Ana Gamberale, Norma González, Domingo Palmero, Silvia Altabe, Norberto Simboli, Noemí Kaoru Yokobori

**Affiliations:** Instituto Nacional de Enfermedades Infecciosas, ANLIS “Dr. C. G. Malbrán,” Buenos Aires, Argentina (R. Paul, F. Lorenzo, B. López, N. Simboli, N.K. Yokobori); Programa de Control de Tuberculosis, Chaco, Argentina (M.G. Alegre, S. Altabe); Université des Antilles, Guadeloupe, France (D. Couvin); Institut Pasteur de Guadeloupe, Abymes, Guadeloupe (D. Couvin, N. Rastogi); Hospital General Universitario Gregorio Marañón, Madrid, Spain (L. Pérez-Lago, D. García de Viedma); Instituto de Investigaciòn Sanitaria Gregorio Marañón, Madrid (L. Pérez-Lago, D. García de Viedma); Instituto de Salud Carlos III, Madrid (D. García de Viedma); CIBER Enfermedades Respiratorias, Madrid (D. García de Viedma); Hospital Muñiz, Buenos Aires (A. Gamberale, D. Palmero); Instituto Vaccarezza, Buenos Aires (A. Gamberale, D. Palmero); Hospital General de Niños “Pedro de Elizalde,” Buenos Aires (N. González); National Scientific and Technical Research Council, Buenos Aires (N.K. Yokobori)

**Keywords:** Tuberculosis, multidrug resistant, pre-extensively drug-resistant, extensively drug-resistant, bedaquiline, linezolid, X family, bacterial fitness, tuberculosis and other mycobacteria, Argentina, antimicrobial resistance, *Suggested citation for this article*: Paul R, Lorenzo F, López B, Alegre MG, Couvin D, Rastogi N, et al. Outbreak caused by multidrug-resistant *Mycobacterium tuberculosis* with unusual combination of resistance mutations, northern Argentina, 2006–2022. Emerg Infect Dis. 2025 Mar [*date cited*]. https://doi.org/10.3201/eid3103.241272

## Abstract

To reconstruct transmission chains of the multidrug-resistant tuberculosis Ch strain, which harbors a unique combination of resistance mutations, we analyzed genomes of 25 isolates from 12 patients with diagnosis during 2006–2022 in Chaco Province, Argentina. Amplification of resistance, high mortality rates, and indications of a wider outbreak raise concerns for surveillance programs.

Argentina is considered a mid-incidence country for tuberculosis (TB); 1% of multidrug-resistant (MDR) cases persist in Argentina. The northern province of Chaco, a province with a top 5 TB burden (*1*), had a low number of MDR TB cases and no prior evidence of local transmission (*2*). MDR TB can affect patients beyond the well-established risk groups, making clinical suspicion essential where universal drug-susceptibility testing (DST) is unavailable. Whole-genome sequencing (WGS) enables timely and precise molecular drug-resistance profiling, but genotype/phenotype correlations need further research, especially for rapidly emerging resistance to second-line drugs (e.g., bedaquiline and linezolid) (*3*). 

To reconstruct the transmission chains and drug-resistance profiles, we used WGS to analyze an MDR TB outbreak in Resistencia, Chaco Province, Argentina. The study was performed in accordance with the Helsinki Declaration as revised in 2013. The Research Ethics Committee of the Instituto Nacional de Epidemiologia, ANLIS “Dr. Jara,” Buenos Aires, Argentina, approved the project and waived the informed consent requirement (project code YOKOBORI05/2022).

## The Study

During 2018–2019, our laboratory received 9 MDR *M. tuberculosis* isolates from 3 patients of Resistencia, Chaco, that had unexpectedly inconsistent PCR-based resistance profiles for rifampin and isoniazid in isolates from the same patient and between 2 patients with close epidemiologic links (Appendix 1; Appendix2). A preliminary genomic analysis showed simultaneous presence of the mutations *rpoB*_Asp435His, *rpoB*_His445Asp, *fabG1-inhA*_t-8c, and *katG*_Ser315Thr. We studied that possible outbreak because of the unusual combination of mutations, the extended resistance profile, and the critical disease in young patients with no relevant comorbidities (Table 1).

**Table 1:** Characteristics of patients at time of diagnosis of MDR-TB infection with Ch strain, Argentina, 2006–2022*

ID	Residence	Year	Age, y/sex	DR profile†	DR status	HIV status	Comments
1	Resistencia, Chaco	2006	57/M	STR, INH, RIF, EMB, PZA, ETH	MDR	Unknown	Index case-patient. No previous treatment records. Died 2 months after diagnosis. Treatment was with INH, RIF, PZA, EMB, and STR.
2	Resistencia, Chaco	2008	NA/F	STR, INH, RIF, EMB, PZA, ETH	MDR	Negative	Mother of patients 3, 4, and 9. Worked as a nurse at hospital where patient 1 was assisted. Self-administered the second-line drugs. Smear positive for AFB until 2011.
3	Resistencia, Chaco	2010	14/M	STR, INH, RIF, EMB, PZA, ETH (2LI, FLQ)	MDR (pre-XDR)	Negative	Son of patient 2. Hepatotoxicity associated with TB drugs. Poor adherence to treatment. Problematic substance use. Died in 2016.
4	Resistencia, Chaco	2010	16/M	STR, INH, RIF, EMB, PZA, ETH	MDR	Negative	Son of patient 2. Hepatotoxicity associated with TB drugs.
5	Resistencia, Chaco	2014	66/M	STR, INH, RIF, EMB, PZA, ETH	Pre-XDR	Unknown	Grandfather of patient 6. Repeated treatment changes, suboptimal treatment.
6	Resistencia, Chaco	2014	15/F	STR, INH, RIF, EMB, PZA, ETH, FLQ (LZD‡)	Pre-XDR (XDR)	Negative	Granddaughter of patient 5. Her mother, who had lupus, and brother died of TB (Appendix 2}. Discharged in 2016 after sputum tested negative. Relapsed in 2017. Referred to a hospital that specialized in DR-TB in Buenos Aires in 2019. Poor adherence to treatment. Smoker.
7	Corrientes, Corrientes	2014	52/M	STR, INH, RIF, EMB, PZA, ETH	MDR	Positive	Died in 2015.
8	Resistencia, Chaco	2017	43/F	STR, INH, RIF, EMB, PZA, ETH	MDR	Unknown	TB diagnosed in 2016. Had contact with a TB patient in 1999, but the association with the outbreak is unknown.
9	Resistencia, Chaco	2017	18/F	STR, INH, RIF, EMB, PZA, ETH (CAP, ETH)	MDR	Negative	Daughter of patient 2. Irregular treatment and poor adherence. Died in 2021.
10	Resistencia, Chaco	2018	16/F	STR, INH, RIF, EMB, PZA, ETH, FLQ (CFZ, BDQ§)	Pre-XDR (XDR)	Negative	Friend of patient 6, who visited her frequently. Repeated treatment changes. Admitted to a pediatric hospital in Buenos Aires in 2019, where she received CFZ/BDQ. Patient complied with treatment; her condition improved, and she was discharged in December 2019. Relapsed and died in 2020 during social isolation because of COVID-19 pandemic.
11	Resistencia, Chaco	2020	24/F	STR, INH, RIF, EMB, PZA, ETH	MDR	Negative	Irregular treatment. Problematic substance use.
12	Del Viso, Buenos Aires	2021	21/M	STR, INH, RIF, EMB, PZA, ETH	MDR	Unknown	Patient was unavailable for follow-up until mid-2022 when he started second-line treatment. Former resident of Resistencia and declared the same address of patient 2 and her family, but their exact relationship is unknown.

*BDQ, bedaquiline; CAP, capreomycin; CFZ, clofazimine; DR, drug resistance; EMB, ethambutol; ETH, ethionamide; FLQ, fluoroquinolone; INH, isoniazid; NA, not available; MDR, multidrug-resistant; pre-XDR, pre–extensively drug-resistant; PZA, pyrazinamide; RIF, rifampin; STR, streptomycin; XDR, extensively drug-resistant.; 2LI, second-line injectable drugs. †Consolidated DR profiles based on phenotypic and molecular test results. STR was tested phenotypically in all isolates until 2017. The DR profile and DR status of the last isolate are indicated between brackets. ‡Confirmed phenotypically. §Tested retrospectively after the detection of the *Rv0678*_114duplC mutation by whole-genome sequencing.

The Mycobacteria Service, Instituto Nacional de Enfermedades Infecciosas, ANLIS “Dr. C. Malbrán,” Buenos Aires, Argentina, the national reference laboratory for TB diagnosis, has kept an MDR genotyping database, representing nationwide cases, since 2003. After intense screening of our databases, on the basis of rifampin and isoniazid molecular resistance patterns; province of origin; epidemiologic link, genotype, or both, we identified 29 candidate isolates from 12 patients. Twenty-four isolates were available for WGS analysis (Appendix 1); we assigned an identification number to each isolate. We performed phenotypic DST when we received the isolates unless otherwise stated (Appendixes 1, 2), and we compared spoligotypes with those in the SITVITEXTED database (Appendix 3). 

All patients were residents of Resistencia, except for a patient from the neighboring Corrientes city and a former resident of Resistencia who received their diagnoses in Buenos Aires (Table 1; Appendix 3). Patient 2, a healthcare worker at the hospital where patient 1 was assisted, was the mother of patients 3, 4, and 9. Patient 6 was the granddaughter of patient 5 and frequently visited her friend, patient 10. The remaining epidemiologic links were unknown. Five patients were teenagers at the time of diagnosis (Table 1).

**Figure 1:**
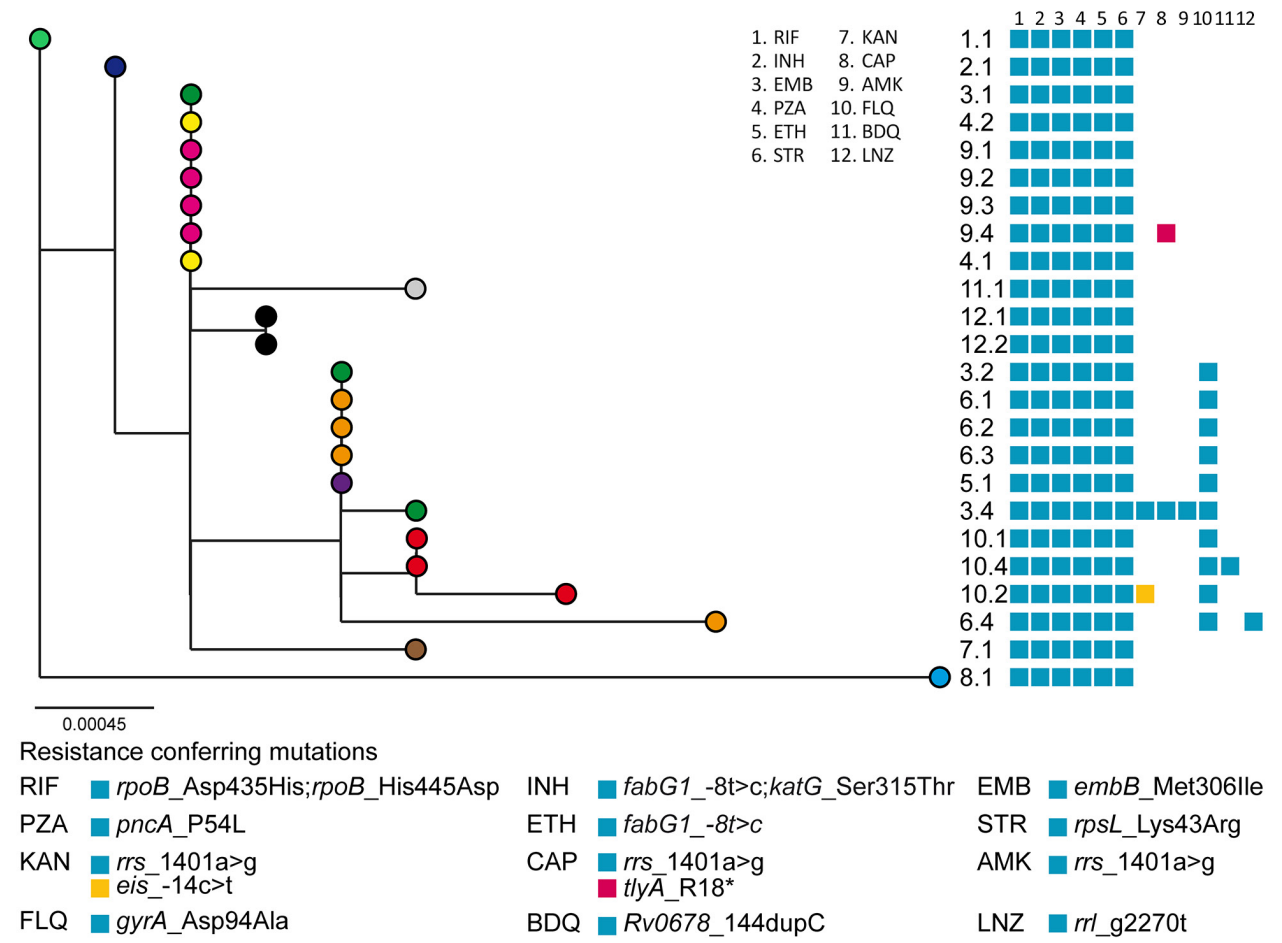
Maximum-likelihood phylogenetic tree of the 24 isolates from the MDR *Mycobacterium tuberculosis* outbreak strain Ch, Chaco, Argentina, 2006–2022, analyzed by whole-genome sequencing. Each patient is represented by a colored dot. Labels indicate the patient and isolate identification numbers. Blocks indicate the resistance-conferring mutations detected in each isolate, as indicated below the tree. Scale bar indicates number of substitutions per variable site. MDR, multidrug-resistant.

WGS showed a monophyletic group (Figure 1; Appendix 2). We named the clone the Ch strain, and it belonged to lineage 4.1.1 and to the spoligotype international type (SIT) 119 of the X1 family. The highest number of SIT119 clones in the SITVITEXTEND database in the region, without association with MDR, is in Brazil (Appendix 3). The median pairwise single-nucleotide polymorphism distance among the first isolates from each patient was 3 (range 0–6) (Appendix 2), excluding isolate 8.1, which was 15 single-nucleotide polymorphisms (range 12–17) apart from the others. The most ancestral isolate belonged to the index case-patient, whose diagnosis was made in 2006. Despite the patient having no history of TB treatment, that isolate had the 8 resistance mutations common to the cluster, including the double mutations for isoniazid and rifampin (Table 2; Figure 1), suggesting that the outbreak could have been more extended. Patient 3 was probably the source of 3 secondary pre–extensively drug-resistant cases in the second subcluster. Isolates from the patients with the most recent diagnoses (patients 11 and 12) were closer to the first subcluster (Figure 1).

**Table 2:** Resistance conferring mutations found in Ch strain isolates from patients with MDR-TB infection, Argentina, 2006–2022*

Mutation	Drug	Confidence grading	Phenotype/genotype†	Concordance
*rpoB_*Asp435His	RIF	Associated with resistance–interim	24/24	Yes
*rpoB_*His445Asp	RIF	Associated with resistance	24/24	Yes
*fabG1-inhA_*t-8c	INH	Associated with resistance–interim	24/24	Yes
	ETH	Associated with resistance–interim	3/24‡	Partial
*katG_*Ser315Thr	INH	Associated with resistance	24/24	Yes
*embB*_Met306Ile	EMB	Associated with resistance	18/24§	Partial
*embA*_c-11a	EMB	Uncertain significance	NA	NA
*pncA*_Pro54Leu	PZA	Associated with resistance	24/24	Yes
*rpsL*_Lys43Arg	STR	Associated with resistance	24/24	Yes
*rrs*_a1401 g	KAN	Associated with resistance	1/1	Yes
	AMK	Associated with resistance	1/1	Yes
	CAP	Associated with resistance	1/1	Yes
*eis*_c-14t	KAN	Associated with resistance	1/1	Yes
	AMK	Associated with resistance–interim	0/1	No
	CAP	NA	0/1	NA
*tlyA*_Arg18stop (LoF)	KAN	NA	0/1	NA
	AMK	NA	0/1	NA
	CAP	Associated with resistance	1/1	Yes
*gyrA*_Asp94Ala	FLQ	Associated with resistance	10/10¶	Yes
*rrl*_g2270t	LZD	Associated with resistance–interim	1/1#	Yes
*Rv0678*_144dupC (LoF)	BDQ	Associated with resistance	1/1	Yes
	CFZ	Associated with resistance	1/1**	Yes

*Confidence grading according to the World Health Organization Catalogue of Mutations, 2nd Edition (*3*). AMK, amikacin; BDQ, bedaquiline; CAP, capreomycin; CFZ, clofazimine; EMB, ethambutol; ETH, ethionamide; FLQ, fluoroquinolone; INH, isoniazid; LoF, loss of function; LZD, linezolid; NA, not applicable; PZA, pyrazinamide; RIF, rifampin; Stop, stop codon; STR, streptomycin; 2LI, second-line injectable drugs**.**†For the isolates studied by whole-genome sequencing; phenotypically resistant isolates/isolates harboring each mutation are indicated. ‡Fifteen isolates were sensitive, 5 isolates had discordant results between methods, and 1 isolate was not tested. §Two isolates were susceptible, 3 had discordant results between proportion and MGIT methods, and 1 isolate was not studied. ¶Resistance to low dose of moxifloxacin. #Isolate 6.4 had a MIC of 4 mg/L for LZD (suggested cutoff value 0.5 mg/L). **Isolate 10.4 had a MIC of 2 mg/L for CFZ (suggested cutoff value 0.25 mg/L). Phenotypic resistance to BDQ was implemented in 2021, and isolate 10.4. was tested in the light of the genomic results.

**Figure 2:**
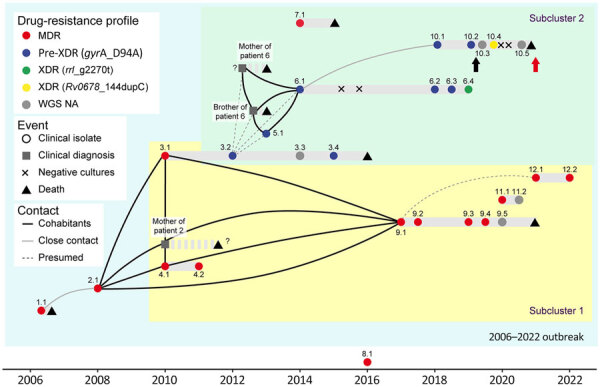
Schematic representations of the timeline and epidemiologic links of the MDR *Mycobacterium tuberculosis* outbreak with strain Ch, Chaco, Argentina, 2006–2022. Two subclusters were defined according to the phylogenetic analysis (Figure 1). The symbols represent epidemiologic events. Patients represented with squares were identified as part of the outbreak after comprehensive epidemiologic research in the light of the genomic results (Appendix 2). They had received a clinical diagnosis, and the drug susceptibility profiles were not available. Dot colors represent the drug-resistance status and the mutation found. The exact date of the events indicated with a question mark is unknown. Black arrow indicates administration of bedaquiline to patient 10, and the red arrow indicates implementation of phenotypic drug-susceptibility testing for that drug in Argentina. MDR, multidrug resistant; WGS NA, whole-genome sequence not available; XDR, extensively multidrug resistant.

The resistance mutations had high World Health Organization (WHO) confidence gradings and were concordant with the phenotypic drug-susceptibility testing results (Table 2), except for ethionamide and ethambutol, as expected (*3*). Resistance to the second-line injectable drugs was acquired independently in 3 isolates (Figure 1). Two isolates were extensively drug resistant. Isolate 6.4 had a *rrl*_g2270t mutation, which has recently been associated with linezolid resistance (*3*), and isolate 10.4 had the loss-of-function mutation *Rv0678_*144dupC. Resistance to linezolid and cross-resistance to bedaquiline were confirmed by phenotypic methods (Table 2). Instances of clofazimine/bedaquiline resistance were acquired shortly after their administration under strict supervision (Figure 2).

No compensatory mutations were found in *rpoC* or *rpoA*. Because both mutations in *rpoB* are expected to have a mild to high fitness cost (*4*), their simultaneous presence could constitute a unique compensatory mechanism.

Patient 1 died shortly after diagnosis. The mother of patient 2 had received a TB diagnosis in 2010 but refused treatment and died in 2011. The mother and the brother of patient 6 died in 2013 (Appendix 2). Isolates from those patients were not available. Because isolates 3.2, 5.1, and 6.1 were identical (Figure 1), the other 2 relatives could have acquired their infection from patient 3 (Figure 2), but their epidemiologic link remains unknown. Their close relationship, the contemporaneity, and their fatal outcomes strongly suggest that those patients were part of the outbreak (Figure 2). Patient 9 probably experienced relapse from latency and infected patient 12, who lived at the same address before moving to Buenos Aires. Their relationship remains unclear. Patient 2 administered the medication to her family, including the injectable drugs. Some regimens, especially in the first years of the outbreak, were suboptimal and underwent multiple changes. Reports suggest that empathy from some healthcare providers had been insufficient. Three cohabitants received chemoprophylaxis with isoniazid, and no active disease has been reported (Appendix 2).

## Conclusions

The MDR Ch outbreak strain, with its epicenter in Resistencia, Chaco, belonged to the X1, SIT 119 spoligotype, which is infrequent in Argentina (*5*,*6*) (Appendix 3). The X family has been associated with high transmissibility (*7*,*8*), and SIT119 could have been imported from Brazil, considering the frequent cross-border movements to and from Chaco (Appendix 3). Further phylogenomic studies are warranted to determine the precise origin of that MDR strain.

Several factors converged in the outbreak. It started as intrahospital transmission to a healthcare worker, who spread the disease to her cohabitants (Figure 2). Despite her background, the family was poorly receptive to and not compliant with treatment. On the other hand, some healthcare workers were not appropriately prepared to manage MDR TB. Delayed diagnosis, poor compliance, and administration of suboptimal regimens led to long periods of culture positivity, amplification of resistance, and an alarmingly high mortality rate (53%). In addition to lack of effective drugs, host and bacterial genetic factors could have played a role. Several patients were relatives, which suggests genetic susceptibility to TB. Conversely, the observed mortality rate suggests higher virulence of the Ch strain. Although challenging to assess their relative effect, those biological and societal factors collectively shaped the outbreak outcome.

We gained valuable insights from our study. The phylogenetic analysis strongly suggests additional missing cases, and although no new cases were diagnosed, MDR TB surveillance in Chaco must be strengthened. Of note, Ch genotype can be suspected by detecting the *rpoB* double mutation through GeneXpert and other molecular tests (data not shown). Next-generation tools, including novel drugs and WGS, are available, but clinical suspicion of MDR-TB remains crucial for their effective use. Continuing education and active engagement of healthcare professionals and the community are vital for managing future outbreaks.

Appendix 1 Supplementary methods for study of multidrug-resistant tuberculosis outbreak with unusual combination of resistance mutations, northern Argentina, 2006–2022.

Appendix 2 Characteristics of isolates from study of multidrug-resistant tuberculosis outbreak with unusual combination of resistance mutations, northern Argentina, 2006–2022.

Appendix 3 Distribution of SIT119/X1 and X family strains in SITVITEXTEND.
