# Supplementary material for: Outbreak Caused by Multidrug-Resistant Mycobacterium Tuberculosis with Unusual Combination of Resistance Mutations, Northern Argentina, 2006–2022
Source: Emerg Infect Dis. 2025 Mar;31(3):601–6. doi: 10.3201/eid3103.241272 (PMC11878304; doi:10.3201/eid3103.241272)
Supplement: Appendix 1 — Supplementary methods for study of multidrug-resistant tuberculosis outbreak with unusual combination of resistance mutations, northern Argentina, 2006–2022. [file 24-1272-Techapp-s1.pdf]

Article DOI: https://doi.org/10.3201/eid3103.241272
EID cannot ensure accessibility for supplementary materials supplied by authors. Readers who have difficulty accessing supplementary content should contact the authors for assistance.

Outbreak Caused by Multidrug-Resistant *Mycobacterium tuberculosis* with Unusual Combination of Resistance Mutations, Northern Argentina, 2006–2022

Appendix 1

Supplementary Methods

In Argentina, genotyping of MDR *Mycobacterium tuberculosis* has been implemented in mid-1990s. MDR-TB has been systematically surveyed since 2003, and as a best practice, it is highly encouraged that all the isolates detected countrywide are sent to the *Servicio de Micobacterias, INEI, ANLIS* for second-line drug-susceptibility testing and genotyping at referral level. Our laboratory keeps a genotyping database of MDR isolates evaluated with *IS6110*-RFLP and spoligotyping (1998–2010, ~1400 isolates), MIRU-VNTR15 (2012–2020, ~1050 isolates) or WGS (2021 onwards, ~400 isolates), and these isolates are stored at −80°C. Since 2012, all the *M. tuberculosis* isolates that are suspected of drug-resistance have been routinely tested in our lab with an in-house PCR-based test named MAS-PCR (*1*), which has been internally validated. MAS-PCR targets the wild type *rpoB* (codons 452, 450, 445 and 435), *fabG1-inhA* operon (−15 position) and *katG* (codon 315) genes to infer the resistance to RIF and INH. The results are stored in a tailored database (>6000 isolates). Epidemiologic and microbiological information associated with each isolate are stored in an internal database.

We exhaustively screened these databases to select isolates potentially belonging to the outbreak, based on province of residence, rifampin (RIF) and isoniazid (INH) molecular resistance patterns, genotype, microbiological and epidemiologic data. Twenty-nine isolates from 12 patients received between 2006 and 2022 were included and re-cultured from our collection.

Twenty-four genomes were included in the final analysis (Appendix Figure). The detailed information associated with each of the isolates is included in Appendix 2 (https://wwwnc.cdc.gov/EID/article/31/3/24-1272-App2.xlsx).

Epidemiologic information was complemented by the TB control program of Chaco, the *Hospital Muñiz/Instituto Vaccarezza* and the *Hospital General de Niños "Pedro de Elizalde."*

Phenotypic drug-susceptibility testing

The following drugs were evaluated with the MGIT960 system: RIF (critical concentration: 0.5 mg/L), INH (0.1 mg/L), ethambutol (EMB; 5mg/L), ethionamide (ETH; 5mg/L), kanamycin (KAN; 2.5mg/L), capreomycin (CAP; 2.5mg/L), amikacin (AMK; 1mg/L), levofloxacin (LFX; 1mg/L), clofazimine (CFZ; 1mg/L), linezolid (LZD; 1mg/L), moxifloxacin (MXF) at low (0.25 mg/L) and high concentrations (1 mg/L). Resistance to EMB (2 mg/L), ETH (40 mg/L) and CAP (40 mg/L) were also assessed by the proportion method in Löwenstein-Jensen medium. Some isolates were tested for LZD and CFZ resistance by the microtiter dilution (6 serial 1:2 dilutions starting from 8 mg/L) following standardized methods (*2*). Suggested ECOFF for LZD and CFZ were of 0.5 mg/L and 0.25 mg/L respectively (*3*,*4*). Phenotypic susceptibility test for bedaquiline (BDQ) was implemented in 2021 in MGIT960 (critical concentration: 1mg/L).

Whole genome sequencing analysis

The selected isolates were re-cultured from our collection in Löwenstein-Jensen slants. High quality DNA was recovered from 23 isolates by the CTAB method (*5*). Whole genomes of isolates 6.2 and 6.3 had been obtained previously for another project (*6*) and were retrieved from the ENA (PRJEB41837). The genome of isolate 12.2 had been sequenced for referential diagnosis and was included in the second edition of the Catalogue of mutations of WHO (*7*). Genomic libraries were prepared using the DNA Sample Preparation Kit and indexed with the Barcoding Kit (Illumina), following the instructions of the manufacturer. Isolates were sequenced in the Illumina MiSeq or Novaseq platform at the *Unidad Operativa Centro Nacional de Genómica y Bioinformática*, ANLIS, with paired-end mode. Quality assessment included analysis with *FastQC* v0.12.1. Average sequencing depth was 80x. Sequencing reads were aligned to the H37Rv genome using *BWA* 0.7.17 (*8*). Allelic variants were called with *GATK* 4.4.0.0 (*9*) and were annotated with *snippy* (*10*). Variants in PE/PPE genes, repetitive regions

and mobile elements were excluded. Maximum likelihood phylogenetic trees were constructed with *RAxML* (*11*) and visualized with *Microreact* (*12*). Drug-resistance conferring mutations were detected with *Mykrobe* (*13*) and *TB-profiler* version 6.2 (*14*) and were contrasted with the 2nd version of the WHO catalog of mutations (*7*). Spoligotypes were retrieved in silico using *TB-profiler*. One genome had a low QC value and was discarded (Supplementary Figure. 1.1). Accession numbers of the genomes are detailed in Appendix 2. SIT119 isolates were searched in the SITVITEXTEND database hosted at the Pasteur Institute of Guadeloupe (Appendix 3, https://wwwnc.cdc.gov/EID/article/31/3/24-1272-App1.pdf).

References

1. Yang Z, Durmaz R, Yang D, Gunal S, Zhang L, Foxman B, et al. Simultaneous detection of isoniazid, rifampin, and ethambutol resistance of Mycobacterium tuberculosis by a single multiplex allele-specific polymerase chain reaction (PCR) assay. Diagn Microbiol Infect Dis. 2005;53:201–8. PubMed https://doi.org/10.1016/j.diagmicrobio.2005.06.007

2. Clinical and Laboratory Standards Institute. M24 Susceptibility Testing of Mycobacteria, Nocardia spp., and Other Aerobic Actinomycetes, 3rd ed. Wayne, PA: The Institute; 2018.

3. Lopez B, Siqueira de Oliveira R, Pinhata JMW, Chimara E, Pacheco Ascencio E, Puyén Guerra ZM, et al. Bedaquiline and linezolid MIC distributions and epidemiological cut-off values for *Mycobacterium tubercu*losis in the Latin American region. J Antimicrob Chemother. 2019;74:373–9. PubMed https://doi.org/10.1093/jac/dky414

4. Schön T, Juréen P, Chryssanthou E, Giske CG, Sturegård E, Kahlmeter G, et al. Wild-type distributions of seven oral second-line drugs against *Mycobacterium tuberculosis*. Int J Tuberc Lung Dis. 2011;15:502–9.

5. van Soolingen D, Hermans PW, de Haas PE, Soll DR, van Embden JD. Occurrence and stability of insertion sequences in *Mycobacterium tuberculosis* complex strains: evaluation of an insertion sequence-dependent DNA polymorphism as a tool in the epidemiology of tuberculosis. J Clin Microbiol. 1991;29:2578–86. PubMed https://doi.org/10.1128/jcm.29.11.2578-2586.1991

6. Pérez-Lago L, Monteserin J, Paul R, Maus SR, Yokobori N, Herranz M, et al. Recurrences of multidrug-resistant tuberculosis: Strains involved, within-host diversity, and fine-tuned allocation of reinfections. Transbound Emerg Dis. 2021;69:327–36.

7. World Health Organization. Catalogue of mutations in *Mycobacterium tuberculosis* complex and their association with drug resistance. 2nd ed. Geneva: The Organization; 2023.

8. Li H, Durbin R. Fast and accurate short read alignment with Burrows-Wheeler transform. Bioinformatics. 2009;25:1754–60. PubMed https://doi.org/10.1093/bioinformatics/btp324

9. Broad Institute. Gatk [cited 2023 Aug 30]. https://github.com/broadinstitute/gatk/releases

10. Seemann T. Snippy [cited 2023 Aug 30]. https://github.com/tseemann/snippy

11. Stamatakis A. RAxML version 8: a tool for phylogenetic analysis and post-analysis of large phylogenies. Bioinformatics. 2014;30:1312–3. PubMed https://doi.org/10.1093/bioinformatics/btu033

12. Argimón S, Abudahab K, Goater RJE, Fedosejev A, Bhai J, Glasner C, et al. Microreact: visualizing and sharing data for genomic epidemiology and phylogeography. Microb Genom. 2016;2:e000093. PubMed https://doi.org/10.1099/mgen.0.000093

13. Hunt M, Bradley P, Lapierre SG, Heys S, Thomsit M, Hall MB, et al. Antibiotic resistance prediction for *Mycobacterium tuberculosis* from genome sequence data with mykrobe. Wellcome Open Research. 2019;4. https://doi.org/10.12688/wellcomeopenres.15603.1

14. Phelan J. jodyphelan/TBProfiler [cited 2023 Aug 30]. https://github.com/jodyphelan/TBProfiler

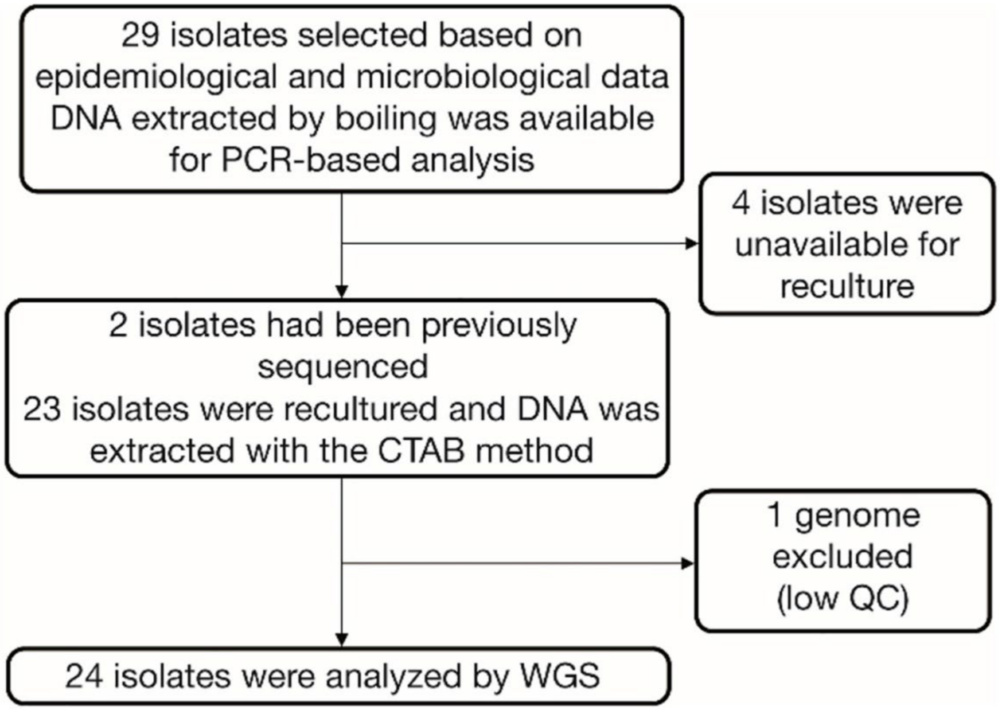

Appendix 1 Figure. Inclusion and exclusion criteria.
