# Supplementary material for: Outbreak Caused by Multidrug-Resistant Mycobacterium Tuberculosis with Unusual Combination of Resistance Mutations, Northern Argentina, 2006–2022
Source: Emerg Infect Dis. 2025 Mar;31(3):601–6. doi: 10.3201/eid3103.241272 (PMC11878304; doi:10.3201/eid3103.241272)
Supplement: Appendix 3 — Distribution of SIT119/X1 and X family strains in SITVITEXTEND. [file 24-1272-Techapp-s3.pdf]

Article DOI: https://doi.org/10.3201/eid3103.241272
EID cannot ensure accessibility for supplementary materials supplied by authors. Readers who have difficulty accessing supplementary content should contact the authors for assistance.

Outbreak Caused by Multidrug-Resistant *Mycobacterium tuberculosis* with Unusual Combination of Resistance Mutations, Northern Argentina, 2006–2022

Appendix 3

Distribution of SIT119/X1 and X family strains in SITVITEXTEND

Spoligotype International Type (SIT)119/X1 strains and X family strains in SITVITEXTEND database were analyzed. Regarding the worldwide distribution of the SIT119/X1 spoligotyping profile, it can be noted that this profile was predominant (>1.00%) in 10 countries, including South American countries such as Brazil, Peru and Colombia (Appendix 3 Table 1).

When we look at the global distribution of X family strains, we see that Brazil and Peru are among the 10 countries with the highest number of X family strains (Appendix 3 Table 2).

A Maximum Likelihood (ML; Appendix 3 Figure 1) phylogenetic tree and a Minimum Spanning Tree (MST; Appendix 3 Figure 2) were constructed based on spoligotyping patterns belonging to X family and representing at least 0.1% of strains in SITVITEXTEND database. SIT numbers were used to identify the nodes. These figures (Appendix 3 Figures 1, 2) showed that SIT119/X1 occupied a central position in each tree, indicating that it is an ancestral spoligotype, with a single spacer loss compared to SIT53.

Isolates belonging to SIT119 or X1 clade, other than those included in this study, are rare in the Argentinean databases (Appendix 3 Table 3). Our MIRU-VNTR15 database was screened using the MIRU-VNTR plus platform but additional isolates potentially belonging to SIT119 were not found. This database contains three X family strains and two of them

were beloged to SIT119 isolates from Germany, but no closely related genotypes, other than the Ch strain, were found. The closest SITs (e.g., 217, 336, 490, 878, etc.; Appendix 3 Figure 2) are also unusual.

Peruvian migrants are infrequently found in Chaco. Residents from Chaco frequently go to the Paraguay-Brazil-Argentina triple border and other southern Brazilian cities for business or tourism. The closest border is shared with Paraguay and has a constant movement of people with Chaco, but X family strains are rare in this country (https://doi.org/10.1186/1471-2180-7-75).

SIT119 isolates from Brazil and Peru, were not associated with MDR (Appendix 3 Table 3) and acquisition of MDR status of the Ch strain in Chaco could not be discarded.

Appendix 3 Table 1. Distribution of SIT119/X1 in SITVITEXTEND (showing countries with global percentage >1.00%).

Country Name	Number	Global percentage of SIT119/X1 (%)
United States	630	55.75
South Africa	143	12.65
Mexico	104	9.20
Brazil	34	3.01
United Kingdom	28	2.48
India	22	1.95
Netherlands	16	1.42
Peru	14	1.24
Colombia	12	1.06
Italy	12	1.06

Appendix 3 Table 2. Distribution of X family strains in SITVITEXTEND (showing countries with global percentage >1.00%).

Country Name	Number	Global percentage of X family (%)
United States	2580	50.55
South Africa	655	12.83
Brazil	239	4.68
United Kingdom	181	3.55
Mexico	179	3.51
Peru	179	3.51
Haiti	101	1.98
Sweden	59	1.16
Spain	54	1.06
India	54	1.06

Appendix 3 Table 3. X1 isolates, other than the Ch strain, identified in Argentina. 1995 – 2009 and 2021 - 2023

No.	Year	Province	Method	DR profile	SIT	Clade	Genotype	Comment
1	1995	Buenos Aires	RFLP, spoligtyping	INH mono resistant	119	X1	Unique	No additional information available
2	2008	Buenos Aires	RFLP, spoligotyping	Susceptible	119	X1	Unique	No additional information available
3	2021	Buenos Aires	WGS	MDR	119	X1	Unique	Foreign born. Treatment failure.
4	2023	Buenos Aires	WGS	RR	119	X1	Unique	No additional information available
7	1997	Buenos Aires	RFLP, spoligtyping	Susceptible	1080	X1	Unique	No additional information available
8	2007	Córdoba	RFLP, spoligtyping	MDR	1080	X1	Unique	Patient born in Peru.
9	2007	Buenos Aires	RFLP, spoligtyping	Susceptible	1080	X1	Unique	Health care worker
10	2009	Santa Fe	RFLP, spoligtyping	MDR	1080	X1	Unique	Person deprived of liberty
11	2022	Buenos Aires	WGS	MDR	1080	X1	Unique	No additional information available

No.	Year	Province	Method	DR profile	SIT	Clade	Genotype	Comment
5	2023	Santa Fe	WGS	INH mono resistant	1080	X1	Cluster 1	No additional information available
6	2023	Santa Fe	WGS	INH mono resistant	1080	X1	Cluster 1	No additional information available
12	2022	Buenos Aires	WGS	Susceptible	2250	X1	Unique	No additional information available
13	2022	Córdoba	WGS	Susceptible	ORPH	X1	Cluster 2	No additional information available
14	2023	Buenos Aires	WGS	Susceptible	ORPH	X1	Cluster 2	Foreign born.

WGS clusters were defined using a SNP threshold of 20. The relatedness among RFLP defined genotypes and WGS is unknown and unique genotypes were defined for each technique. ORPH: 777756777760731.

Appendix 3 Table 4. Distribution of drug resistance profiles for SIT119/X1 and X family in Argentina, Brazil and Peru (based on the SITVITEXTEND database)

		N of isolates (%)		
Isolate	Profile	Argentina	Brazil	Peru
SIT119/X1	Pansusceptible (code 1)	1 (100.00)	11 (32.35)	13 (92.86)
	MDR (code 2)	0 (0.00)	0 (0.00)	0 (0.00)
	Resistance to any drug (code 3)	0 (0.00)	2 (5.88)	0 (0.00)
	XDR (code 4)	0 (0.00)	0 (0.00)	0 (0.00)
	Unknown (code 0)	0 (0.00)	21 (61.76)	1 (7.14)
X family	Pansusceptible (code 1)	6 (27.27)	42 (17.57)	143 (79.89)
	MDR (code 2)	0 (0.00)	34 (14.23)	13 (7.26)
	Resistance to any drug (code 3)	1 (4.55)	18 (7.53)	11 (6.15)
	XDR (code 4)	0 (0.00)	0 (0.00)	2 (1.12)
	Unknown (code 0)	15 (68.18)	145 (60.67)	10 (5.59)

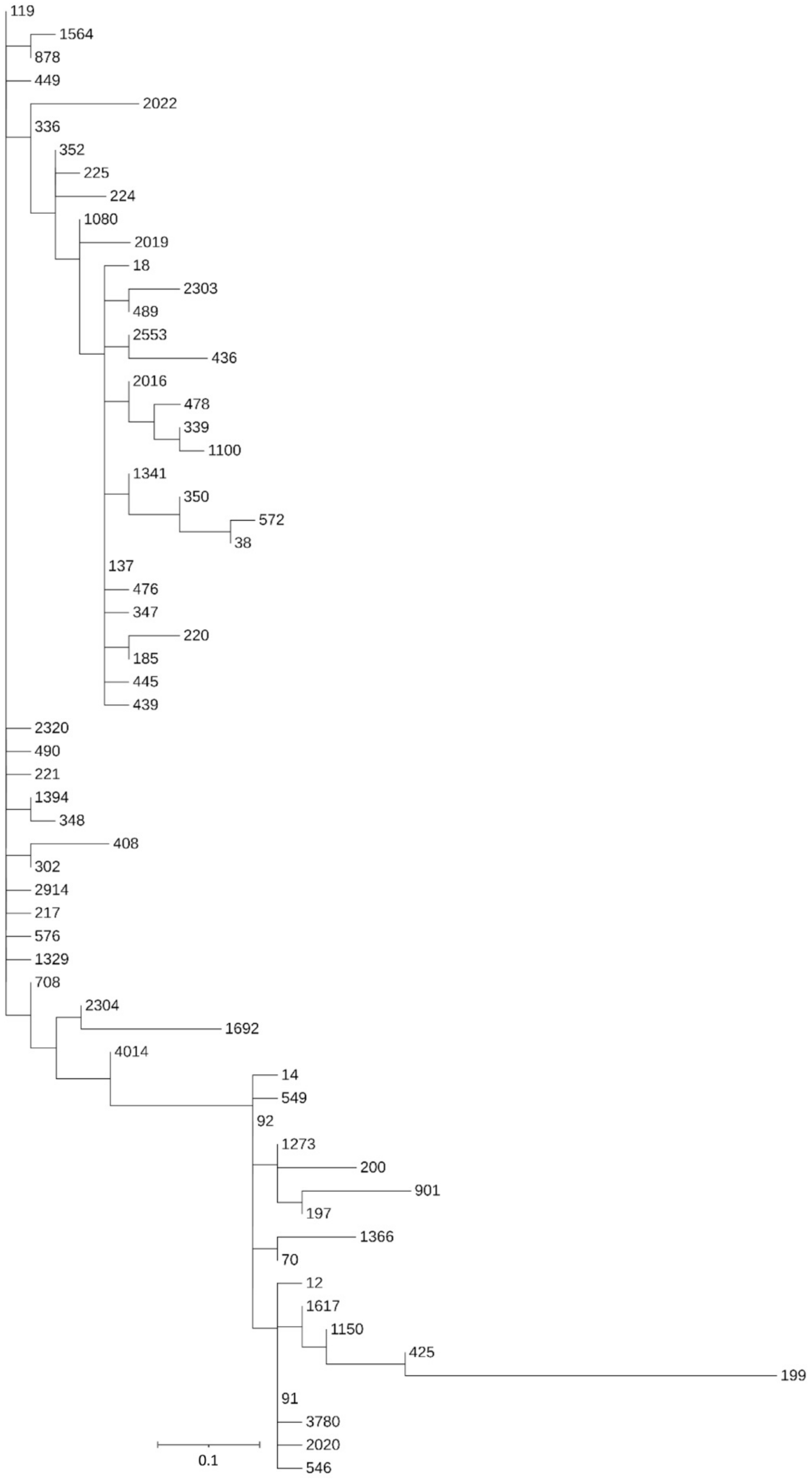

Appendix 3 Figure 1. Maximum Likelihood phylogenetic tree drawn using IQ-TREE software, based on spoligotyping patterns (binary format). SIT numbers (representing >0.1% of the strains of family X) are indicated as nodes of the tree.

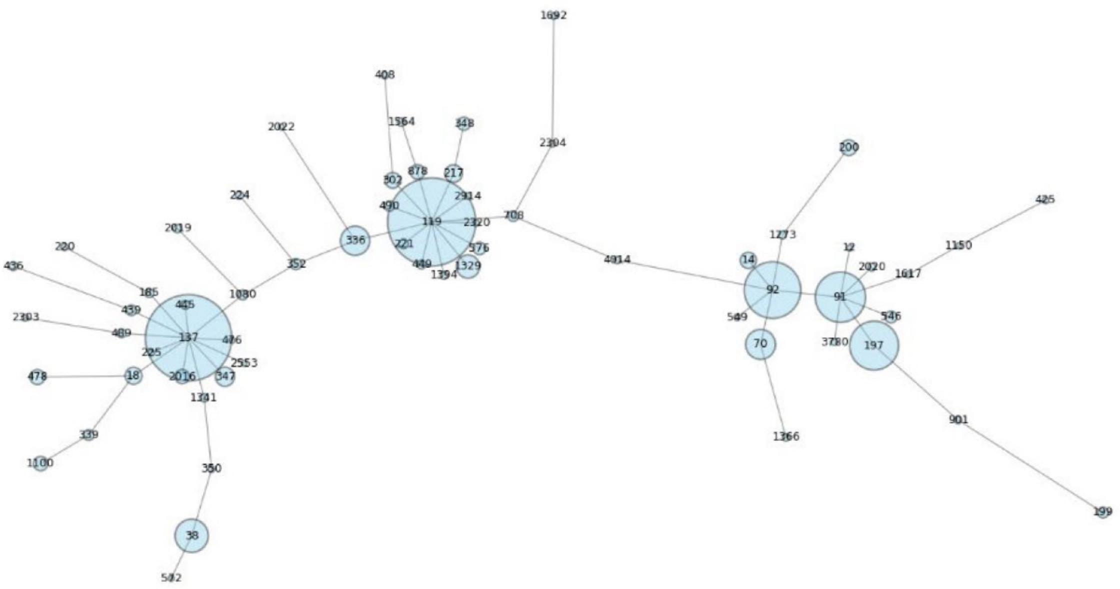

Appendix 3 Figure 2. Minimum Spanning Tree (MST) based on Kamada Kawai's algorithm (used from a custom Python script), showing SIT numbers representing >0.1% of the strains of family X in SITVITEXTEND. The size of the circle is proportional to the number of strains for each SIT.

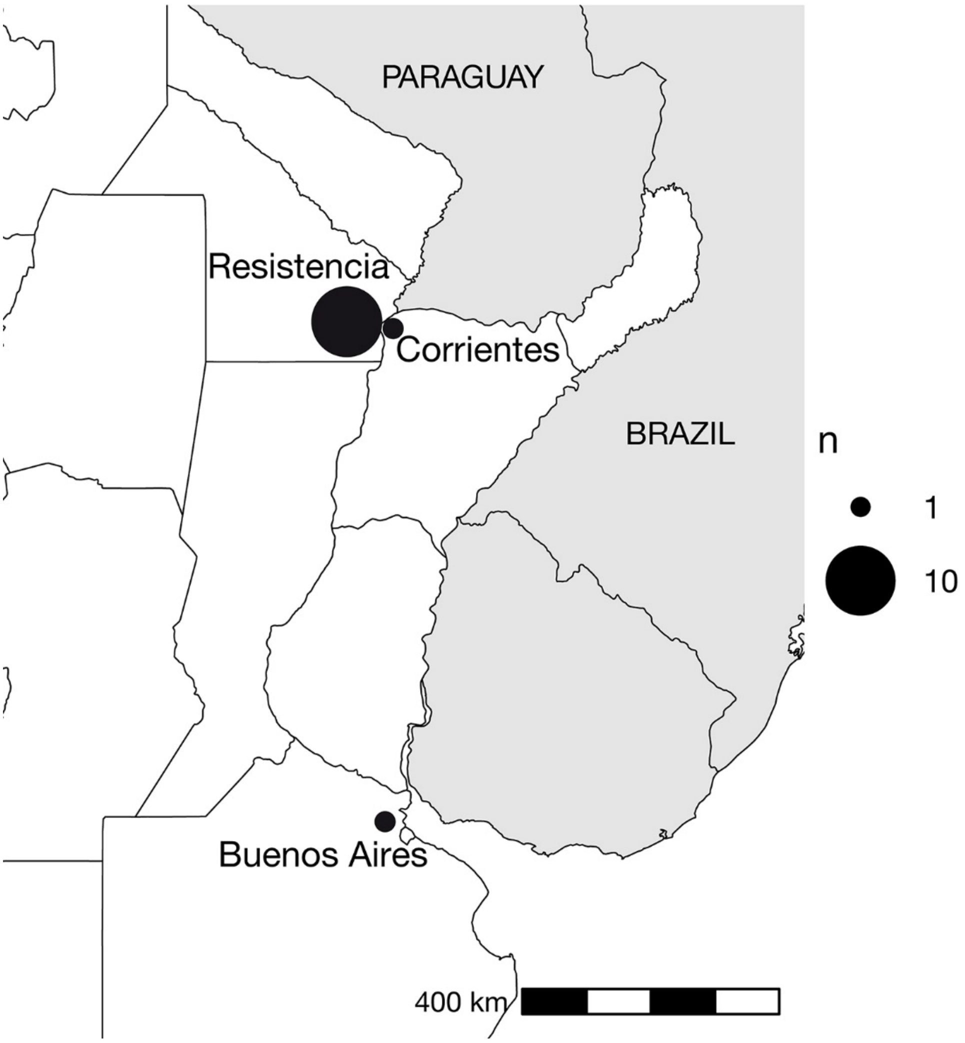

Appendix 3 Figure 3. Geographic distribution of Ch strain cases.
